# Promotion Policies for Electric Vehicle Diffusion in China Considering Dynamic Consumer Preferences: A Network-Based Evolutionary Analysis

**DOI:** 10.3390/ijerph19095290

**Published:** 2022-04-26

**Authors:** Ruguo Fan, Rongkai Chen

**Affiliations:** School of Economics and Management, Wuhan University, Wuhan 430072, China; rgfan@whu.edu.cn

**Keywords:** electric vehicles, environmental policy, consumer preference, evolutionary game theory

## Abstract

An improved understanding of how policies can promote the diffusion of electric vehicles (EVs) is critical to achieving sustainable development. Previous studies of EV diffusion dynamics have paid insufficient attention to consumer preferences. In this paper, a network-based evolutionary game model considering dynamic consumer preference is constructed to study EV diffusion. Through numerical experiments, the evolutionary processes and results of various promotion policies, including carbon taxes, production subsidies, purchase subsidies, and information policy on EV diffusion, are simulated. In particular, this paper explores the differentiated effects of supply-side policies and demand-side policies. The simulation results indicate that: (1) The effectiveness of promotion policies is sensitive to the size of the manufacturer network, and large networks can dampen periodical fluctuations in diffusion rates. (2) Supply-side carbon taxes and subsidies facilitate a steady diffusion of EVs. However, compared with the sustained effectiveness of subsidies, carbon taxes may inhibit the rapid penetration of EVs. (3) Implementing purchase subsidies in the early stages of diffusion is more effective than production subsidies, but the potential uncertainty of demand-side subsidies should be noted. (4) The impact of information policy on the evolutionary trend of EV diffusion is pronounced but is a longer-term impact, requiring a long enough implementation horizon.

## 1. Introduction

The transportation sector, an important pillar of socioeconomic development, is energy-intensive and accounts for approximately 30% of total global energy consumption [1]. With global population growth and economic activities expanding, transportation-related energy demand is predicted to continue to grow. In particular, energy consumption due to passenger vehicles is expected to show strong growth against the background of rapid urbanization in emerging economies. However, environmental pollution and energy shortages created by the transportation sector are becoming increasingly prominent and may further exacerbate the systemic risks of climate change [2]. Promoting the diffusion of electric vehicles (EVs) and thus replacing internal combustion engines vehicles (ICEVs) has been widely recognized as a promising solution to address these issues and achieve sustainable development [3].

The diffusion of EV faces many economic and technical barriers, including high upfront costs, technological externalities, and lower consumer preference, making the EV diffusion relatively slow and limited to niche markets. As governments have implemented various EV-related policies to address market failures and promote EV diffusion, understanding and comparing the effects of different policy levers are critical to instituting improved policies to achieve the target of large-scale EV penetration.

The effect of promotion policy on the diffusion of EV technology among manufacturers is the focus of a growing body of literature. Many recent studies have investigated the dynamic diffusion of EVs among manufacturers and the effects of various policy interventions. For example, Li et al. [4] used a network-based evolutionary game model to study the dynamic influence of tax, subsidy, and license restriction policies on EV diffusion. Hu et al. [5] constructed an evolutionary game model in a small-world network to explore the impact of policies such as subsidies, travel restrictions, and infrastructure construction on manufacturers’ adoption of EV technology. Zhao et al. [6] utilized a three-stage evolutionary game model to study the impact of government subsidies on the diffusion of new-energy vehicles under different network structures.

Previous studies have provided valuable insights into the dynamic process of EV diffusion and the effects of policy interventions on technology diffusion among manufacturers. However, most of them have focused only on the complex interactions between manufacturers in the market and have treated consumers as an exogenous factor in the technology diffusion system. In other words, existing EV diffusion studies tend to model technology diffusion only among manufacturers on the supply side, without considering consumers on the market demand side and their dynamic characteristics. From the perspective of market supply and demand, the static consumer preferences imply that a fixed percentage of consumers repeatedly buy a certain product each year, which not only omits the reality of evolving consumer preferences, but also neglects relevant feedback mechanisms, such as the periodical fluctuations of the market. Both manufacturers and consumers are an integral part of the automotive market, and many theoretical and empirical studies have proven that market demand is an essential driver of products diffusion [7,8]. Promoting manufacturers to produce EVs alone will not create the market demand generated by consumers purchasing EVs and may even lead to a “rebound effect”. Therefore, for the study of EV diffusion among manufacturers, it is necessary to include consumers as stakeholders, especially those with dynamic evolutionary characteristics.

Regarding the policy effect, many studies have examined EV-related policies using various methodologies, but most of them discuss policies on one side of the market in isolation and do not explore the differentiated impacts of supply-side policies with demand-side policies, leaving a key knowledge gap. Additionally, considering the reality that the policy mix of China’s EV industry has started to shift from being producer-oriented to being consumer-oriented [9], it is worth discussing whether such a policy transition is reasonable and what the differences are between the effects and mechanisms of these two types of policies. Achieving the goal of large-scale EV penetration necessarily requires the joint implementation of both types of policies. Comparing the implementation effects and evolutionary trends of multiple policies on EV diffusion is vital for instituting a well-designed policy package.

In light of the illustrated realities, there is a clear need to delve into the gaps above in the literature. We seek to answer the following questions. First, regarding consumers as stakeholders, how do dynamic consumer preferences affect the diffusion of EVs among manufacturers? Second, how do various policies implemented for different stakeholders affect the trends and results of EV diffusion? Third, what are the differential effects of supply-side and demand-side policies on EV diffusion?

In this paper, we first choose two types of policies, supply-side and demand-side policies, through literature review, and identify the main stakeholders in EV diffusion. Then, we construct a network-based evolutionary game model to study the EV diffusion in the manufacturer network. Subsequently, consumer dynamics within the coordination game framework are introduced on the demand side to extend the EV diffusion model from the perspective of dynamic consumer preferences. Through controlled numerical experiments, we simulate the process and results of EV diffusion under various policies, thus providing further insights into the effects of various policies and their differences and informing the effective implementation of government policies.

Academically, this study contributes to the existing literature in three ways: (1) An EV diffusion model that includes dynamic consumer preferences in the manufacturer network is constructed to fill the research gap in the previous literature that treats only manufacturers and governments as stakeholders, ignoring consumer behavior. (2) By analyzing the dynamic effects of supply-side and demand-side policies on EV diffusion and comparing the differences in the effects of differently oriented policies, this paper theoretically enriches the insights on different types of interventions. (3) This paper provides a theoretical tool for governments to develop effective policy intervention mechanisms and design an improved policy package.

This paper proceeds as follows: Section 2 conducts a review of relevant literature and then presents the knowledge gap. Section 3 offers the framework of our network-based evolutionary game model, and the model dynamics are detailed. In Section 4, a series of controlled numerical simulations are run to analyze and discuss the effects of various policies. Section 5 elaborates the conclusion and implications.

## 2. Literature Review

The research related to this paper flows into three major streams: the effect of policy on EV diffusion, consumer preferences, and the network-based evolutionary game. In the following subsections, the studies of these three streams are reviewed, and then knowledge gaps are presented.

### 2.1. The Impact of Policies on Electric Vehicle Diffusion

Policy intervention has always been regarded as a powerful means to facilitate the diffusion of EVs [10,11]. The broader literature on technological diffusion suggests a conventional categorization according to policy objectives: supply-side and demand-side policies [12,13]. The supply-side policies, aimed primarily at manufacturers, attempt to reduce barriers to technology introduction and technology output for manufacturers through R&D subsidies and carbon taxes, etc., thereby directly promoting the production of EVs by manufacturers [14]. In this regard, manufacturer subsidies [14], carbon emission trading [15], energy supervision policy [16], and carbon tax [17] are the focus of EV diffusion research.

Comparatively, demand-side policies are mainly a market demand tools designed to create more demand for EVs by stimulating consumers to purchase EVs and thus indirectly promote the diffusion of EVs in the manufacturer network [18]. Many studies have concerned the effects of consumer incentive policies on the EV industry. For example, Sun et al. [14] studied the effect of public subsidies using an agent-based model for the EV industry. Li et al. [19] analyzed the effectiveness of purchase subsidies, popular science propaganda, charge fees, and social discussions on consumer purchase of EVs based on a consumer network model. Li et al. [4] summarized the supply-side and demand-side policies based on China’s policy practice and simulated two demand-side policies, purchase subsidies, and license plate restriction. As such, in accordance with Li et al. [19] and Kong et al. [15], carbon taxes and EV production subsidies (supply-side policies), as well as consumer subsidies and information policy (demand-side policies), were chosen as the focus of our study.

### 2.2. Consumer Preference

Consumer preferences are a crucial element in predicting and analyzing EV diffusion. In previous studies of EV diffusion, the role of consumer preferences is reflected in providing preferences required to support market demand forecasts for new vehicle technologies [20]. An emerging technology such as EV technology may not be accepted by the market during the initial development phase [21]. The evolution of consumer preference for EVs or ICEVs in the market brings about the evolution of potential market demand for alternative products. As more consumers choose EVs and make the decision to purchase them, the demand for EVs will increase, and thus EV manufacturers will earn more profits [22]. In short, consumer preferences determine market demand for different products and therefore “pull” the automotive market toward EV production, which is also known as the “demand-pull” of the market.

Traditionally, consumer preferences are regarded as static limiting analysis of the consequences of a given sequence of parameters in the economics field [23]. With the recognition that consumer preferences for complex technological products may be unstable [24], scholars have further analyzed and concluded that consumer preferences for new vehicle technologies are probably evolving under different market conditions, i.e., preferences are dynamic [20]. Possible reasons for the evolving preferences are changes in the economic and technological environment or the influence of complex social interactions. Many studies have highlighted social interactions as a potential explanation for dynamic consumer preferences [25,26]. This is because interactions with family, friends, or peers, who may have no direct experience with the products or may be familiar with them, are likely to influence consumer preferences [27,28].

Many researchers have also explored the impact of consumer preferences on the adoption or diffusion of vehicle technologies. Oliveira et al. [20] compared the difference between static and dynamic consumer preferences in a system dynamics model of alternative fuel vehicle diffusion, and their results suggest that dynamic consumer preferences have a significant impact on diffusion and that subsidy policies should be designed according to the evolution of consumer preferences. Oryani et al. [29] investigated Iranian consumers’ heterogeneous preferences for EVs using a choice experiment model. Chakraborty et al. [30] studied the plug-in electric vehicle diffusion in California and found that neighborhood effect and workplace effect influence consumer EV purchases. Lee et al. [31] identified the heterogeneity of early adopters in California plug-in electric vehicle market based on the Bass model. Moon et al. [32] analyzed consumers’ perspectives and preferences for hydrogen-powered vehicles. However, research on EV diffusion among manufacturers has only dealt with static consumer preferences, and few of them have extended the discussion of dynamic consumer preferences in modeling exercises, which motivated us to introduce dynamic preferences into our manufacturer evolutionary game framework for EV diffusion.

### 2.3. The Network-Based Evolutionary Game

Evolutionary game theory is a classical approach based on game theory and evolutionary biology theory to study how players adjust their strategic choices in the process of constant interaction and mutual adaptation. It has become an important method for understanding the formation process and interpreting the evolution mechanism of group behavior and social system formation processes [33]. For manufacturer firms, evolutionary game theory is often used to study the game between them and the dynamic evolution of their strategies. In addition, the long-term and dynamic strategic interaction between manufacturers and other stakeholders such as consumers and governments is also an important research direction.

The evolutionary game framework assumes that populations are homogeneous. However, realistic socioeconomic systems may have complex topological and statistical features that may lead to statistically inequivalent interaction probabilities of agents. Therefore, scholars have combined complex network theory, which studies the topology of complex systems, with evolutionary game research and proposes the network-based evolutionary game model [34]. Shi et al. [35] established a network-based evolutionary game model to analyze the impacts of carbon taxes, subsidies, and asymmetric penalties on the diffusion of low-carbon technologies among enterprises. Li et al. [36] applied the well-established PEST framework in a network-based evolutionary game model to identify influencing factors and to study the dynamic impact of these factors on clean energy substitution. Xu et al. [37] constructed an evolutionary game model of manufacturers under government influence based on a scale-free network, and studied the green innovation diffusion mechanism of cloud manufacturing enterprises. Motivated by these preliminary studies, the network-based evolutionary game provides an appropriate approach for capturing the dynamics of EV diffusion in manufacturer network, which is suitable as the basic framework for the study of EV diffusion.

### 2.4. The Gaps

Reviewing the literature on the effects of policies on EV diffusion reveals the following two knowledge gaps.

First, few studies have considered consumers as stakeholders in constructing manufacturer evolutionary game models and considered dynamic consumer preferences on this basis. Although consumers are often modeled as necessary influences on firms’ revenue, most existing evolutionary game models only use the form of the Hotelling demand to calculate the constant product demand corresponding to a certain set of parameters. This implicitly assumes that consumers have constant preferences and purchase the product once a year, which is quite unrealistic for some products, especially EVs. The evolution of EV diffusion and consumer preferences is a process of mutual adaptation and selection, thus simplifying the analysis of consumer preferences would ignore the relevant feedback mechanisms underlying the ultimate diffusion dynamics.

Second, supply-side and demand-side policies are often discussed in isolation, lacking adequate comparison of effects and mechanisms. For policymakers, choosing among various policies entails information about their mechanisms and probable outcomes, particularly in complex economic systems and in situations with far-reaching ramifications. Both supply-side and demand-side policies are vital to EV diffusion, and more comprehensive perception of their effectiveness and mechanisms is needed.

To advance the research streams, this paper proposes a network-based evolutionary game framework for EV diffusion considering dynamic consumer preferences and investigates the effects of different oriented policies on EV diffusion.

## 3. Methods

### 3.1. Problem Description

**Figure 1:**
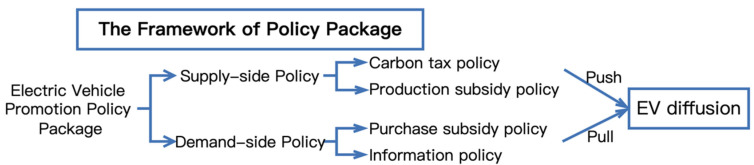
The framework of EV promotion policy package.

The main stakeholders in the EV diffusion process include governments, manufacturers, and consumers. The government implements a series of policies, including supply-side and demand-side policies, to directly or indirectly facilitate manufacturers’ shift towards electric vehicle production. Here, supply-side policies aim to reduce the barriers to technology introduction and output and thus directly facilitate manufacturers’ decisions to produce EVs. In contrast, demand-side policies indirectly promote the diffusion of EVs by influencing consumers’ preferences and purchase decisions for EVs. According to the review of EV diffusion promotion policies in Section 2.1, four policy instruments including carbon tax, EV production subsidy, consumer purchase subsidy, and information policy, are considered and modeled. The framework of the policy package is shown in Figure 1.

**Figure 2:**
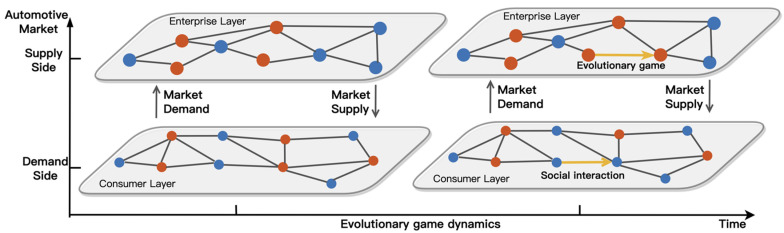
Schematic diagram of the network-based evolutionary dynamics for EV diffusion. The red and blue colors represent manufacturers or consumers who choose different strategies.

Manufacturers are the central players in enabling the diffusion of EVs, and each manufacturer’s decision to produce EVs forms the diffusion of EVs at the market-wide level. This strategic decision comes from the evolutionary game between different agents in the manufacturer network. At the same time, market demand is the foundation for manufacturers to gain revenue. Consumers on the demand side of the market are the demanders of automotive products, and their preferences and purchasing behaviors determine the market demand for EVs as well as ICEVs, thus affecting manufacturers’ payoffs. The evolution of manufacturers’ and consumers’ strategies, shaped by their respective social dynamics, underlies market dynamics at the microlevel. Figure 2 summarizes the schematic diagram of the network-based evolutionary dynamics for EV diffusion.

### 3.2. Modeling Assumptions

Given the above problem description and related literature on EV diffusion, we made the following modeling assumptions for the basis of the network-based evolutionary game model:

1. There are M manufacturers in the automobile market with two pure strategies that are faced with strategic decisions of producing EVs or ICEVs. The initial EV manufacturer proportion is σ(0), while the ICEV manufacturer proportion is 1−σ(0). All the manufacturers are limited rationality and make decisions independently and synchronously.
2. The different strategic decisions adopted by manufacturers will have different carbon-reduction rates, r, and carbon abatement costs, C. We assume a quadratic relationship between carbon-reduction rate and carbon-abatement cost, i.e., $C=kr^{2}/2,\;$ where k is the cost coefficient of abatement, and a similar setting is widely used by scholars.
3. The manufacturer agents are embedded in a complex social network, with nodes representing manufacturers and edges representing linkages between manufacturers. Decision and payoff information is shared among connected nodes in the manufacturer’s network.
4. Manufacturers of the same strategic decision produce homogeneous automotive products. The market demand for EVs and ICEVs is supplied evenly by manufacturers producing EVs and ICEVs, respectively.
5. Automotive products have a lifespan, l, and consumers need to buy a new EV or ICEVs after this period of ownership. This process is influenced by the maturity of EV technology.
6. The size of consumers in the market is N. Consumers are divided into two categories: those who choose EVs or those who choose ICEVs, and the initial ratios are denoted by ε(0) and 1 − ε(0), respectively. Each consumer does not own more than one vehicle at each moment.

### 3.3. The Network-Based Evolutionary Game Model

As an important method of combining evolutionary game theory with complex network theory, the construction of a network-based evolutionary game model consists of three main parts: network structure, game model, and evolutionary mechanism. In this paper, a network-based evolutionary game model of EV diffusion is constructed from these three aspects based on the modeling assumptions.

In terms of network structure, manufacturers in the real world are considered to be well-connected, with complex competitive and cooperative relationships and “small world” characteristics. In addition, for the automotive industry, previous research has generally concluded that there is no significant heterogeneity among manufacturers [4,5]. Therefore, the small-world network model is considered to be able to capture the backbone of complex linkages among automobile manufacturers reasonably. Here, we use G=(V, E) to denote the automaker network, where V is the set of nodes and E is the set of undirected edges.

According to the problem description and modeling assumptions, the sales volume of each EV manufacturer and the ICEV manufacturer selling products at time t can be denoted as follows:

(1) $$Q^{e}\left(t\right)=\frac{D^{e}\left(t\right)+N^{e}\left(t\right)}{M\sigma \left(t\right)}$$

(2) $$Q^{f}\left(t\right)=\frac{D^{f}\left(t\right)}{M\left(1-\sigma \left(t\right)\right)}$$

where σ(t) is the ratio of EV manufacturers to total manufacturers at time t. $D^{e}\left(t\right)$ and $D^{f}\left(t\right)$ are the cumulative market demand for EVs and ICEVs, indicating the demand for repeat consumer purchases. $N^{e}\left(t\right)$ denotes the additional demand due to the change in consumer preference for EV at each moment, which is determined by the dynamic consumer preference in Section 3.4.

Furthermore, considering the government’s influence on manufacturers, the government imposes a carbon tax related to carbon emissions on ICEV manufacturers and EV manufacturers, denoted as:

(3) $$t^{f}=ec^{p}Q^{f}\left(t\right)$$

(4) $$t^{e}=\left(1-r\right)ec^{p}Q^{e}\left(t\right)$$

where $c^{p}$ is the carbon price, e is carbon emission from ICEVs, and r is the carbon-reduction rate. The government production subsidy for EV is $v^{e}$. We therefore represent the total profits of EV manufacturers and ICEV manufacturers at time t as follows:

(5) $$W^{e}\left(t\right)=\left(p^{e}-c^{e}+v^{e}\right)*Q^{e}\left(t\right)-t^{e}-kr^{2}/2$$

(6) $$W^{f}\left(t\right)=\left(p^{f}-c^{f}\right)*Q^{f}\left(t\right)-t^{f}$$

where $p^{e}$ and $p^{f}$ are the unit prices of EVs and ICEVs, and $c^{e}$ and $c^{f}$ are the unit production costs of EVs and ICEVs. Then, when two auto manufacturers, denoted as auto manufacturer i and auto manufacturer j, adopt various strategic decisions, the game matrix of the evolutionary game process is as shown in Table 1.

All manufacturers seek to maximize profits, but they cannot directly determine which strategy to adopt using market demand information. They need to observe and compare the strategies adopted by neighboring manufacturers in the network and the corresponding payoffs after each round of the game to conduct social learning and strategy updating. At each step, all manufacturers obtain the payoffs of adopting the corresponding strategies according to the game matrix, and then randomly select a neighbor to compare the profits. When the manufacturer has the same strategy as the chosen neighbor, no strategy change occurs, while the manufacturer with different strategies learns the neighbor’s strategy with the Fermi rule:

(7) $$P_{\left(i\leftarrow j\right)}=\frac{1}{1+\exp\left[\left(W_{i}-W_{j}\right)/k\right]}$$

where the payoffs of manufacturer i and its neighbor j are $W_{i}$ and $W_{j}$, respectively. This evolutionary rule indicates that manufacturers tend to learn the strategies of neighbors with higher payoffs than their own, and the higher the payoffs of their neighbors, the higher the probability of learning. Fermi rule has been widely used as a learning mechanism for agents in previous studies.

### 3.4. The Dynamic Consumer Preference

Inspired by Shi et al. [38] study on the influence of consumer dynamics on low-carbon technologies, we consider the dynamic evolution of consumer groups in EV diffusion. As mentioned earlier, market demand for EVs or ICEVs largely determines the profits of both types of auto manufacturers. Existing research in this area tends to calculate a definite market demand for both types of firms in the form of the Hotelling model, without considering the moment-to-moment changes in market demand in the evolutionary game of firms. Specifically, the diffusion of EV among manufacturers is paralleled by a dynamic evolution of consumer preferences for EVs and their decision on what type of vehicle to purchase. It is the dynamic evolution of consumers that determines the market demand for vehicles at each moment.

Consumer preferences reflect individuals’ love for different products. Preference theory suggests that when people are confronted with different choices, they will evaluate various information to form preferences and make decisions based on the order of preferences. Studies also highlight the deterministic role of utility functions and social interactions on preferences [39]. Since this paper focuses on the evolution of consumer preferences for two products (ICEVs or EVs), it is natural to view consumers as making decision between EVs and ICEVs under the influence of social interaction and utility. We introduce a group behavior mechanism developed within the social coordination game framework to describe dynamic consumer preferences in the market and include it as the market demand component of our network-based evolutionary game model.

In large groups of consumers, everyone’s opinions and decisions are constantly changing as people communicate and influence each other. The evolution of group preferences for EVs is a dynamic process involving a large number of interacting and adaptive consumers. We consider an automotive market with N consumers, where these individuals are also embedded in small-world social network. Each consumer, i, has two alternative strategies, i.e., purchasing an EV or purchasing an ICEV, denoted by $s_{i}\in \left\{+1,-1\right\}$. We assume that consumer utility includes economic utility and social utility, and that economic utility is influenced by the price of the product itself and by government policies. In terms of social utility, consumers’ strategy choices are influenced by all neighboring nodes in the social network, and this influence is reflected in reality by a strong willingness to maintain coordination with others. Social coordination, as an important social interaction, is considered to be prevalent factor for human behaviors. In addition, economic factors are another key factor in determining consumers’ product purchases. According to the studies of Zino et al. [40] and Ye et al. [41], the utility of consumers adopting different strategies can be indicated as follows:

(8) $$U_{i}=\sum_{j\epsilon N\left(i\right)}\left|s_{j}\left(t\right)\right|/d_{i}+u_{e}$$

where $d_{i}$ is the degree of consumer i in the network, N(i) is the set of neighbors adopting the same strategy as consumer i, and $u_{e}$ is the economic utility. The first term is the social utility, calculated by the ratio of neighbors among all neighbors of consumer i who adopt the same strategy as them. Similar utility functions have been widely used in coordination game studies. Regarding economic utility $u_{e}$, we do not focus on differences in the utility of specific consumer purchase strategies but rather on how exogenous policy interventions affect EV diffusion in the presence of consumer dynamics. We therefore set the economic utility of purchasing ICEV to 0 for simplicity. The government may implement the consumer subsidy policy to stimulate consumers to purchase EVs. The consumer subsidy factor, which measures the strength of the consumer subsidy policy, is denoted as $v^{c}$. Then, the economic utility of consumers purchasing EVs is $u_{e}=v^{c}$.

In each round of the manufacturer’s decision evolution, consumers also perform a round of decision evolution. After gaining payoffs, each consumer randomly selects a neighbor in the social network for social learning. Without loss of generality, we also use the Fermi rule as the evolutionary mechanism for the consumer. That is, the larger the payoff difference in social comparison, the more likely consumers are to learn from each other’s strategies. The change in consumer preferences at each moment can be presented as:

(9) $$f^{e}\left(t\right)=\frac{N_{e}\left(t\right)-N_{e}\left(t-1\right)}{N}$$

where $N_{e}\left(t\right)$ is the number of consumers choosing the EV strategy at t. In addition, the technology maturity of EV manufacturers is a key factor influencing consumer decisions. According to Zeng et al. [42], the adoption of emerging technologies by more manufacturers in the market promotes technology maturity and thus influence consumers. We measure technology maturity m(t) using the following equation:

(10) m(t)=m(0)+γ*(1−m(0))*σ(t)

where σ(t) is the ratio of EV manufacturers in Equation (1), and m(0) is the initial technology maturity. γ is the information speed factor which indicates how fast the information on technology maturity among manufacturers affects consumers. A larger γ indicates that consumers are more significantly influenced by the manufacturers’ information. Government can encourage consumers to increase their demand for electric vehicles through information policy. Thus, the market demand for EVs in Equation (1) is modified as follows:

(11) $$Q^{e}\left(t\right)=\frac{m\left(t\right)D^{e}\left(t\right)+Nf^{e}\left(t\right)}{M\sigma \left(t\right)}$$

## 4. Simulation and Discussion

### 4.1. Parameter Initialization Setting

We calibrate our model using data from relevant studies as well as empirical data from China’s automobile industry. In the manufacturer network, the number of manufacturers M is set to 100, 200, and 300, the average degree of the network is 6, and the initial EV manufacturer proportion σ(0) is 0.1 [35]. Regarding sales and costs, the unit selling prices of EV and ICEV are set to 0.3398 and 0.1289 million CNY, and the unit production costs of EV and ICEV are 0.25 and 0.0554 million CNY [43]. Referring to [44], the carbon emissions e of producing an ICEV are 29.7 tons, and the carbon-reduction rate r of producing EVs is 0.105. The cost coefficient of abatement k is 10 [45], the initial technology maturity m(0) is 0.2 [36], and all vehicles have a lifespan of 10 [38]. In terms of consumers, the number of consumers N is 2.1 million CNY, and the proportion of initial EV consumers ε(0) is 0.1 [38].

Based on the above parameter settings, we conduct controlled numerical experiments, and for each policy, the simulation changes only the parameters related to that policy. The simulation of each set of parameters lasts for 100 steps (which can be regarded as 100 years), and the final results are obtained by averaging the results of 100 independent runs to ensure stability. Since this paper focuses on EV diffusion at the system level, the EV diffusion rate, representing the proportion of EV manufacturers to all manufacturers, is of most interest for the simulation experiment.

### 4.2. The Impact of Supply-Side Policies on the Diffusion of EVs

#### 4.2.1. The Impact of Carbon Tax Policy on the Diffusion of EVs

**Figure 3:**
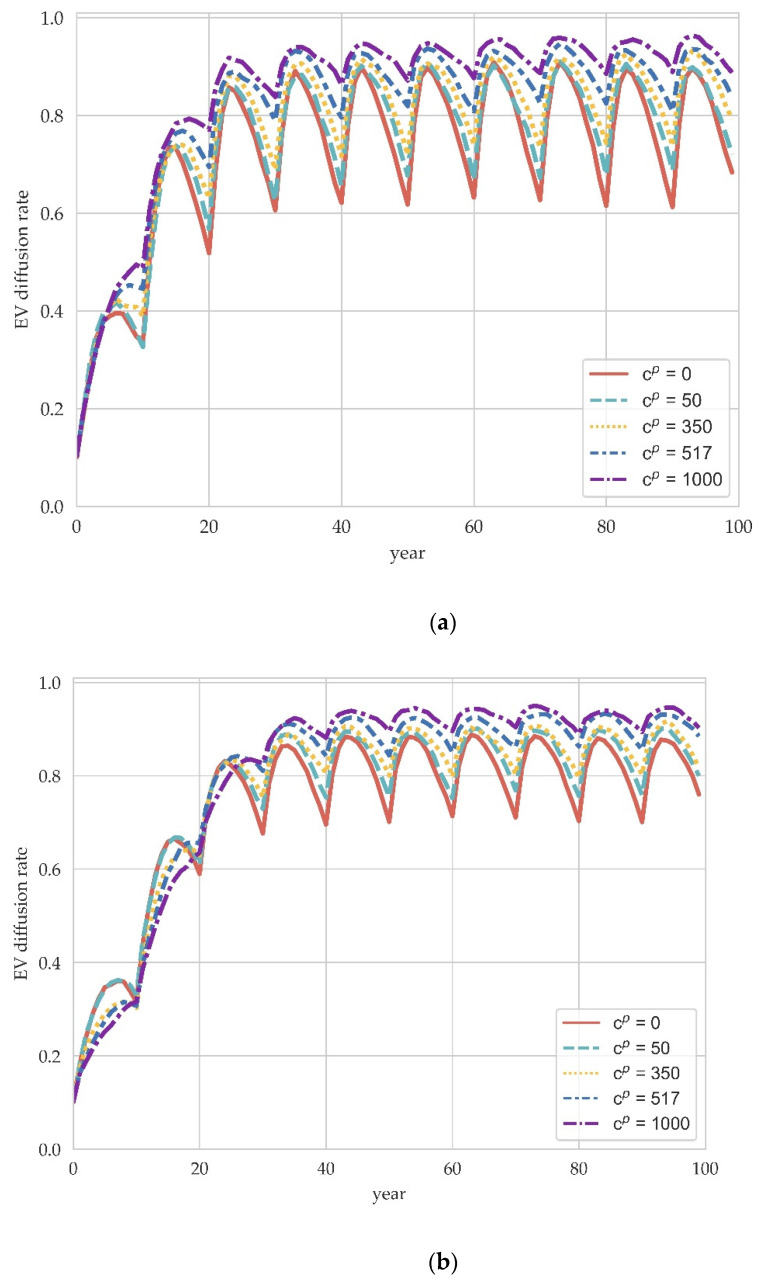

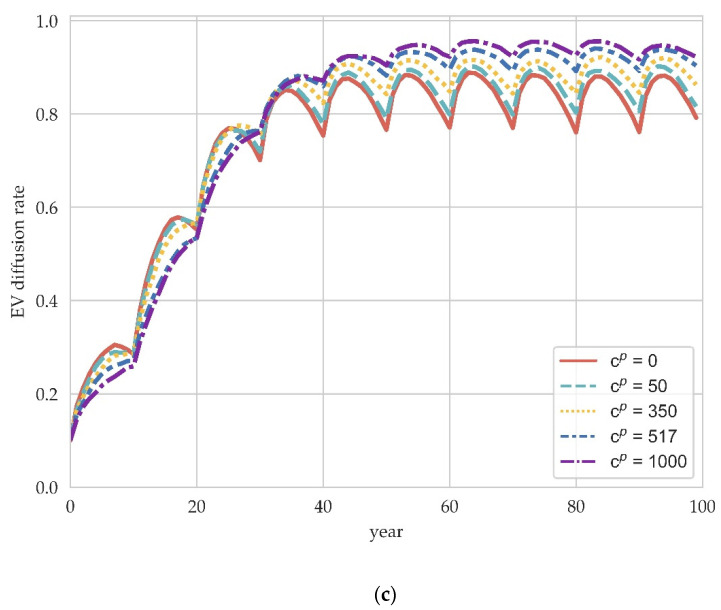
The impact of carbon tax policy on EV diffusion in different scale network. (**a**) M=100. (**b**) M=200. (**c**) M=300.

The carbon tax is charging fees for burning-carbon-based fuels (oil, gas, coal), widely implemented in European countries. In order to study the effect of carbon tax policy on the diffusion of EVs, we conducted simulations at carbon prices $c^{p}$ of 0, 50 (China), 350 (Norway), 517 (Finland), and a fairly high 1000 [38], respectively. The results are shown in Figure 3.

As can be observed from the evolutionary trend, the EV diffusion rate first rises rapidly and then gradually reach equilibrium. The greater the number of manufacturers, the longer it takes to reach equilibrium. For example, it takes about 20 years to reach equilibrium when M=100 and this time extends to about 40 years when M=300. The equilibrium results of the system’s evolution at different carbon prices show that the carbon tax policy favors EV diffusion. At the microlevel, this is because ICEV manufacturers will pay more carbon tax due to more carbon consumption in vehicle production compared with EV manufacturers. The higher carbon tax will reduce the profitability of ICEV manufacturers and thus make them choose to switch to EV production in the long-run evolutionary game.

Additionally, it is worth mentioning that the impact of carbon taxes varies considerably at different evolutionary periods, as shown in Figure 3b,c. Although an increase in the carbon tax at equilibrium increases the EV diffusion rate, this effect seems to be realized after about 35 years. In contrast, a higher carbon price at the stage of rapid EV penetration is detrimental to EV diffusion. This counterintuitive phenomenon may have arisen from the introduction of consumer dynamics. Specifically, the dynamic characteristics of market demand make the evolution of manufacturers largely dependent on the evolution of demand. In the long term, the increasing number of consumers choosing to purchase EVs brings about an increase in the market share of EVs, which is the primary source of continued profit growth for the EV manufacturer population in the market. Additionally, the rate at which such profits rise will determine the rate of EV diffusion. However, during the rapid diffusion stage, a higher carbon tax may reduce the overall profit of manufacturers, thereby inhibiting the social learning mechanism among them. It is not until the equilibrium stage where demand for EVs rises very slowly that the impact of the carbon tax on profit growth becomes negligible. In contrast, the effect on total profits becomes more pronounced. In short, the carbon tax policy has a greater impact on the profit growth rate of EV manufacturers during the rapid diffusion period while on the total profit of ICEV manufacturers during the equilibrium period.

Another interesting finding is that the magnitude of the periodical fluctuation of the EV diffusion rate decreases significantly with the network size increase. When the network size increases from 100 to 300, the maximum diffusion rate during the evolutionary process remains almost unchanged. In contrast, the fluctuation of the diffusion rate in equilibrium decreases by about 2/3. This result indicates that the effectiveness of promotion policies is sensitive to changes in network size. Government policy implementation needs to consider the market environment as well as the number of manufacturers, which is especially important when there are market frictions.

The simulation results support previous studies on the effects of carbon taxes, such as [4,35], which both found that an increase in carbon taxes boosts the diffusion level of products with a competitive advantage. The additional finding of this paper, through the analysis of dynamic consumer preferences, is that the impact of the carbon tax differs across EV diffusion periods, which reflects that a higher carbon tax during the rapid diffusion period reduces the growth rate of diffusion instead. Policymakers need to be cautious in implementing this demand-side policy because tax’s distortion of market adaptive behavior in complex economic systems can be large and unexpected.

#### 4.2.2. The Impact of Production Subsidy Policy on the Diffusion of EVs

**Figure 4:**
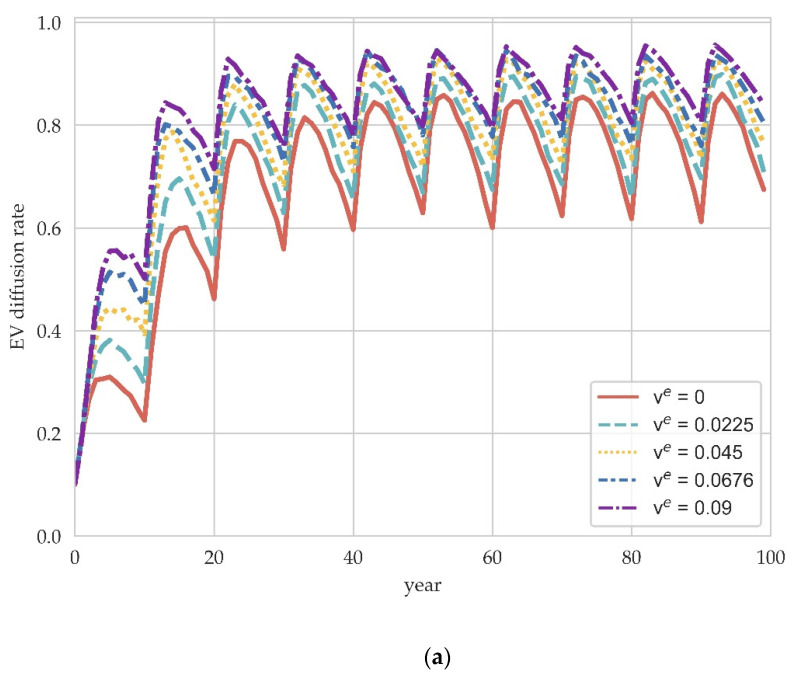

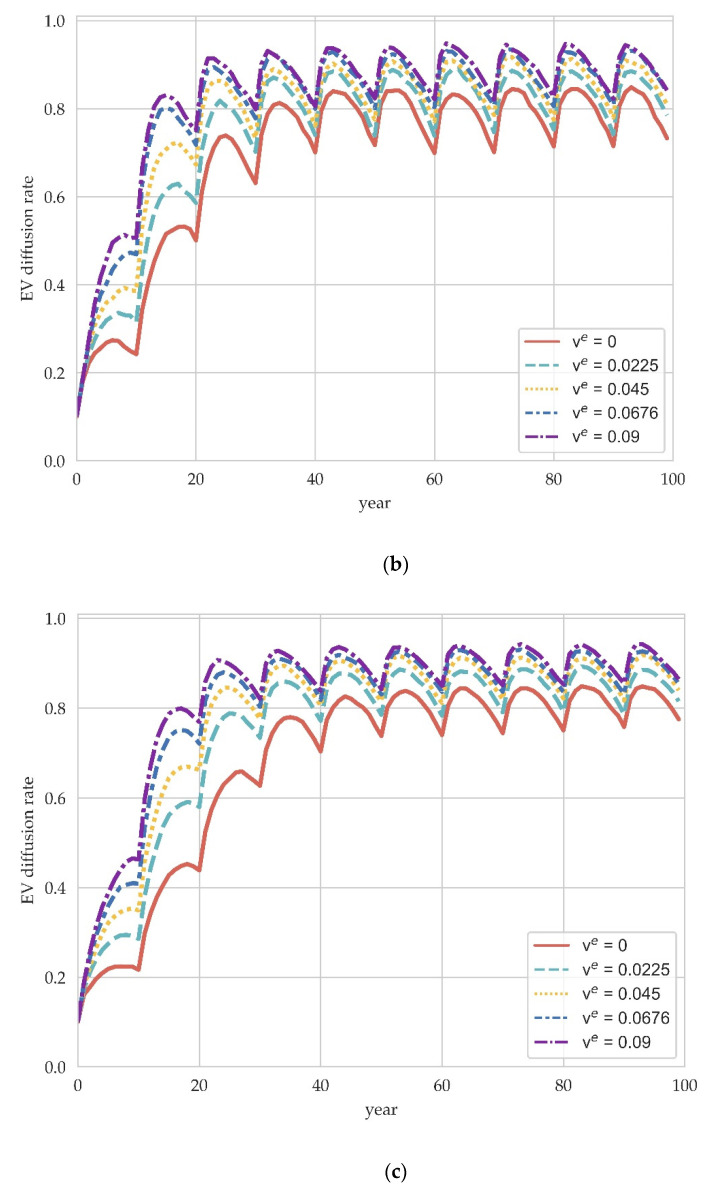
The impact of production subsidy policy on EV diffusion in different scale network. (**a**) M=100. (**b**) M=200. (**c**) M=300.

Subsidies are one of the most common fiscal interventions governments take, and production subsidies are an important source of revenue for EV manufacturers, facilitating the transition to producing EVs. In this section, we illustrate how different production subsidies affect EV diffusion. We run the model with production subsidy $v^{e}$ set to 0, 0.0225, 0.045, 0.0675, and 0.09 [38]. The results are shown in Figure 4.

It can be found that production subsidy policies have a more stable impact on EV diffusion trends and results compared with carbon tax policy. As the production subsidy rises, the diffusion rate increases in any period, which indicates the effectiveness of the supply-side subsidy policy. At the same time, it can be observed that the basic evolutionary trend of EV diffusion is to rise rapidly and then fluctuate at a certain level of diffusion rate, which is in line with the stylized fact of the “S” curve in the traditional innovation diffusion model.

In addition, the model proposed in this paper can capture periodical market fluctuations due to dynamic consumer preferences. Since we set up repeat purchases with product lifespan, this mechanism is reflected at the system level in the diffusion rate fluctuations with a ten-year cycle in Figure 4. This dynamic mechanism is more realistic than constant market demand because the cyclical nature of market demand and the fact that automotive products have a certain life span is self-evident. Indeed, in the field of evolutionary economics, Safarzyńska and van den Bergh [46] also verified the cyclical pattern of demand and the interaction between supply and demand in complex economic systems through an agent-based model. Our findings not only enrich the modeling exercises that consider consumer dynamics in EV diffusion, but also serve as a reminder of the need to factor in potential feedback mechanisms when developing long-term policies.

### 4.3. The Impact of Demand-Side Policies on the Diffusion of EVs

#### 4.3.1. The Impact of Purchase Subsidy Policy on the Diffusion of EVs

**Figure 5:**
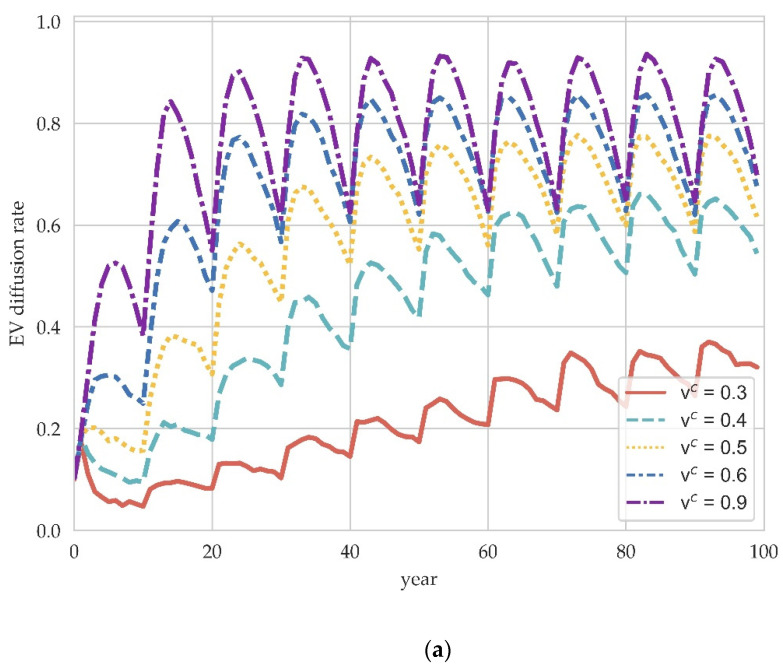

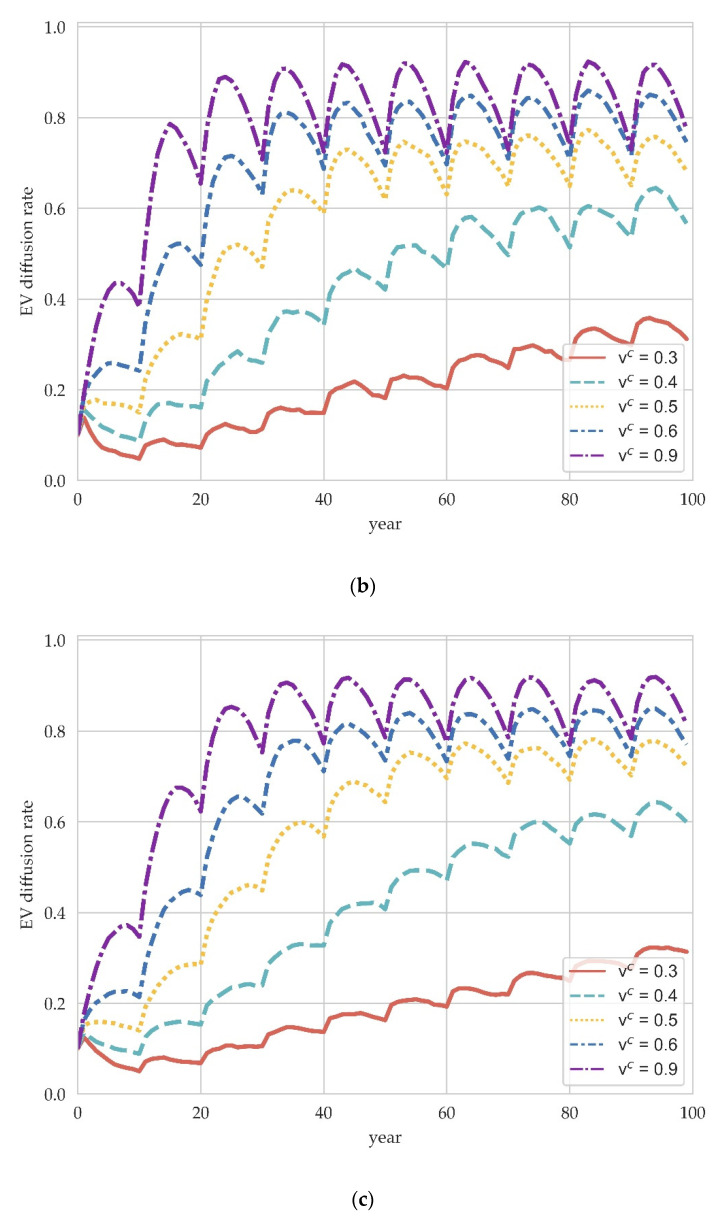
The impact of purchase subsidy policy on EV diffusion in different scale network. (**a**) M=100. (**b**) M=200. (**c**) M=300.

We now elucidate the impact of demand-side policies on the evolutionary trend of EV diffusion and compare the differences in the effects of demand-side and supply-side policies. The effect of consumer subsidies on EV diffusion is given in Figure 5, where the consumer subsidy factors $v^{c}$ are set to 0.3, 0.4, 0.5, 0.6, and 0.9.

Purchase subsidies can be found to play a significant role in the diffusion of EVs. Lower levels of consumer subsidy factors $v^{c}$ may not effectively promote EV diffusion for a long time. In contrast, a higher subsidy factor can achieve EV penetration at a faster rate. The mechanism by which purchase subsidy policy drives EV diffusion is to increase the economic utility of EV consumers, leading to a shift in consumer preferences towards EVs under the influence of social interactions. Furthermore, through the supply–demand interaction in the market, the increase in EV demand improves the profitability of EV manufacturers and hence the popularity of EVs. Studies have proved that purchase subsidies can artificially enhance the attractiveness of EVs, thereby encouraging consumers to become early adopters [14,47].

By comparing the differential effects of supply-side production subsidies and demand-side purchase subsidies, we can find that production subsidies are more influential on long-run diffusion equilibrium, while purchase subsidies have a significant impact on both the diffusion process and equilibrium results. Our modeling exercises to extend consumer dynamics in the manufacturer evolutionary game suggests that the effectiveness of policy interventions on EV diffusion may be sensitive to demand-side dynamics, notably the assumptions about interactions between consumers. For policymakers, this further indicates that demand-side policies may be indispensable and that supply-side efforts alone cannot get rid of consumer dependence on the conventional technology, i.e., the lock-in effect.

In addition, there is greater uncertainty about the effects of purchase subsidies compared to production subsidies. Lower or higher production subsidies would lead to almost only a ten percent difference in effects in equilibrium, while the effects of different purchase subsidy intensities could vary significantly. A possible reason for this phenomenon is the complexity of the economic system. On the one hand, the effects of purchase subsidies are transmitted from the demand side to the supply side through the supply-demand interaction in the market. On the other hand, the demand promotion by purchase subsidies involves complex and highly nonlinear social processes. Together, these factors cause the purchase subsidy to exhibit similar tipping-point dynamics [48]. Therefore, a careful cost–benefit analysis should be conducted when developing subsidy policies, with particular attention to the current status and expectations of EV proliferation. A balance of the appropriate policy mix needs to be undertaken based on the different diffusion targets anticipated and the current diffusion status.

#### 4.3.2. The Impact of Information Policy on the Diffusion of EVs

**Figure 6:**
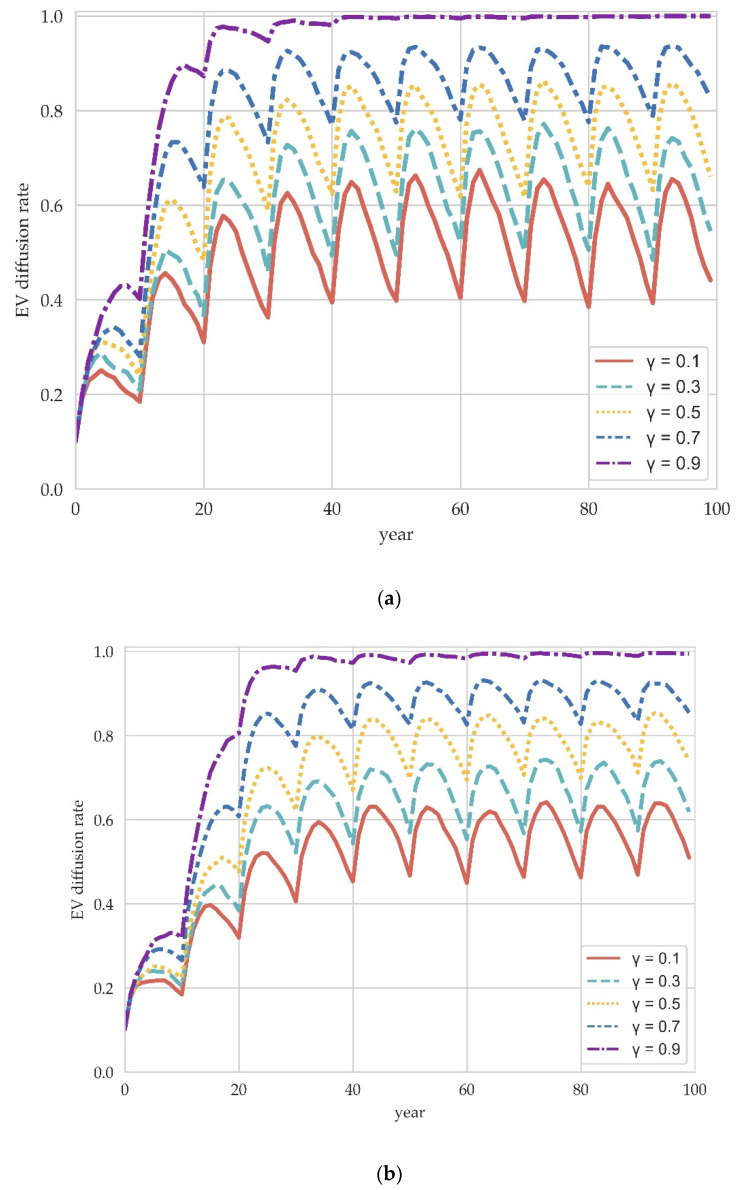

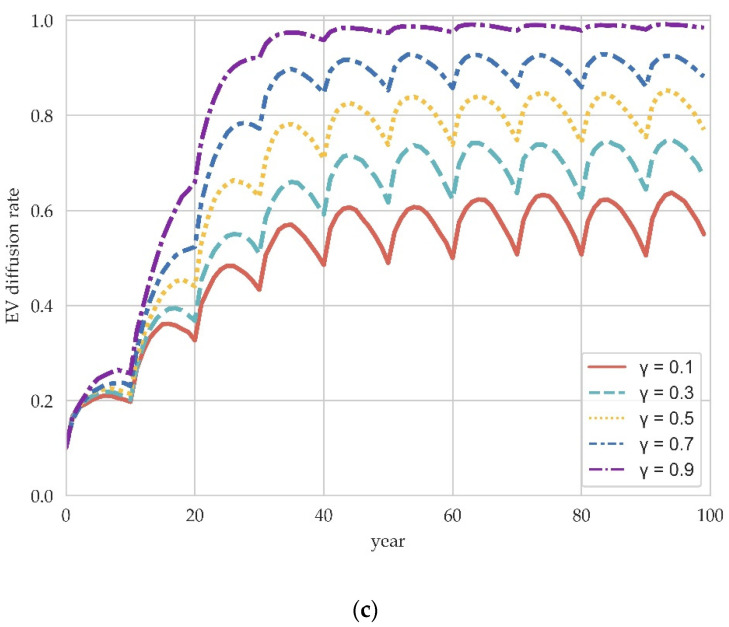
The impact of information policy on EV diffusion in different scale network. (**a**) M=100. (**b**) M=200. (**c**) M=300.

Demand-side information policies that intervene with consumers are gaining attention. Information policies are interventions that reduce negative consumer perceptions of emerging technologies through the Internet or media. We use the information speed factor γ to indicate the degree to which such consumers are influenced by the manufacturer’s information and set γ=0.1, 0.3, 0.5, 0.7 and 0.9 for simulation. The results are shown in Figure 6.

The evolutionary trend shows that the impact of information policy on EV diffusion has been realized gradually over time. There is almost no pronounced effect in the early stages of evolution, while the evolutionary trend becomes markedly different between 10 and 30 years and then fluctuates in equilibrium. This trend is due to the time-lag effect of information policy. Information policy differs from the substantive incentives of purchase subsidies by facilitating information on emerging technologies to be perceived by consumers, thereby promoting repeat purchases and changes in consumer preferences. This process of changing consumer preferences and manufacturer evolution are in a circular cause-and-effect relationship. Improvements in manufacturer technology and the development of the EV industry promote consumer preferences toward EVs through information policy, which in turn contribute to the diffusion of EVs among manufacturers. Furthermore, the comparison with supply-side policies also reveals the longer-term impact of demand-side information policies. Even if information policy does not achieve the same stable promotion effect as production subsidy policy in the short term, the impact on the long-term effect and evolutionary equilibrium is more pronounced.

The longer-term and time-lagged effects of information policy can be found in our results, consistent with a real-world phenomenon in EV diffusion: “hot policies and cold markets” [47]. Despite the government’s efforts to promote and publicize the environmental characteristics and technical performance of EVs, the EV market remains relatively cold. Our results suggest that information policies should be implemented as part of the policy mix over a long enough horizon to achieve the desired effect.

## 5. Conclusions

The large-scale diffusion of EVs is crucial to address environmental pollution and energy shortages. Current research focuses on the evolutionary game of EV production among auto manufacturers while treating consumers as an exogenous factor to the diffusion system. Given the interdependence of market supply and demand and the significant role of consumer preferences, we introduce dynamic consumer preferences into the demand-side modeling of EV diffusion. We propose a network-based evolutionary game framework of EV diffusion considering dynamic consumer preferences, and analyze the differential effects of supply-side and demand-side policies through simulation experiments. The following conclusions can be drawn from the detailed analysis above.

1. The effect of EV promotion policies is sensitive to the size of the network. The increase in the number of manufacturers in the network reduces the magnitude of periodic fluctuations in EV diffusion rates.
2. Both carbon tax policy and production subsidy policy acting on manufacturers can steadily contribute to maintaining a higher level of EV diffusion in equilibrium. However, unlike the effectiveness of production subsidies in the whole diffusion process, carbon tax policy has some inhibiting effects in the phase of rapid diffusion.
3. Demand-side purchase subsidy policy is critical to EV diffusion. Especially in the initial stage of diffusion, implementing the purchase subsidy policy to create market demand is a more appropriate promotion policy. Attention should also be paid to the greater uncertainty of purchase subsidy policies due to the complexity of the economic system. The government’s promotion policy package needs to be adapted and changed according to the expected diffusion targets and the current diffusion stage.
4. The impact of information policy on EV diffusion is pronounced, but its policy effects are longer-term and time-lagged. A long-enough horizon of implementation is needed to ensure the effectiveness of the information policy.

This paper considers dynamic consumer preferences in a model of EV diffusion among manufacturers and conducts simulation experiments for typical supply-side and demand-side policies. Through the in-depth analysis of the mechanisms, evolutionary trends, and equilibrium results of different policies on EV diffusion, this paper not only enriches the modeling practice of EV diffusion, but also provides insights for adjusting the policy orientation and balancing the policy packages. Due to the complexity of the EV diffusion problem, some limitations still exist in this paper. In terms of consumer behavior, the reasons that consumers purchase EVs is quite complex, while this paper only considers social utility under social norms and economic utility under subsidy policies. Future research can introduce more influential factors, such as environmental awareness [49] and consumer attitudes [19], into the demand-side dynamics. In addition, some more complex, agent-based models can also be used to study dynamic consumer preferences and to construct the demand–supply coevolutionary framework.

## Figures and Tables

**Table 1:** The game matrix of manufacturers.

		Auto Manufacturer *j*	
		EV	ICEV
Auto manufacturer *i*	EV	$W^{e}$ $;\;W^{e}$	$W^{e}$ $;\;W^{f}$
	ICEV	$W^{f}$ $,\;W^{e}$	$W^{f}$ $;\;W^{f}$

## References

[1] Li J., Liang M., Cheng W., Wang S. (2021). Life cycle cost of conventional, battery electric, and fuel cell electric vehicles considering traffic and environmental policies in China. Int. J. Hydrogen Energy.

[2] Lu T., Yao E., Jin F., Yang Y. (2022). Analysis of incentive policies for electric vehicle adoptions after the abolishment of purchase subsidy policy. Energy.

[3] Kumar R.R., Guha P., Chakraborty A. (2021). Comparative assessment and selection of electric vehicle diffusion models: A global outlook. Energy.

[4] Li J., Jiao J., Tang Y. (2019). An evolutionary analysis on the effect of government policies on electric vehicle diffusion in complex network. Energy Policy.

[5] Hu Y., Wang Z., Li X. (2020). Impact of policies on electric vehicle diffusion: An evolutionary game of small world network analysis. J. Clean. Prod..

[6] Zhao D., Ji S.-F., Wang H.-P., Jiang L.-W. (2021). How do government subsidies promote new energy vehicle diffusion in the complex network context? A three-stage evolutionary game model. Energy.

[7] Gao X., Rai V. (2019). Local demand-pull policy and energy innovation: Evidence from the solar photovoltaic market in China. Energy Policy.

[8] Guerzoni M., Raiteri E. (2015). Demand-side vs. supply-side technology policies: Hidden treatment and new empirical evidence on the policy mix. Res. Policy.

[9] Xu L., Su J. (2016). From government to market and from producer to consumer: Transition of policy mix towards clean mobility in China. Energy Policy.

[10] Lewis J.I., Nemet G.F. (2021). Assessing learning in low carbon technologies: Toward a more comprehensive approach. WIREs Clim. Chang..

[11] Li W., Long R., Chen H., Dou B., Chen F., Zheng X., He Z. (2020). Public Preference for Electric Vehicle Incentive Policies in China: A Conjoint Analysis. Int. J. Environ. Res. Public Health.

[12] Hoppmann J., Wu G., Johnson J. (2021). The impact of demand-pull and technology-push policies on firms’ knowledge search. Technol. Forecast. Soc. Chang..

[13] Samant S., Thakur-Wernz P., Hatfield D.E. (2020). Does the focus of renewable energy policy impact the nature of innovation? Evidence from emerging economies. Energy Policy.

[14] Sun X., Liu X., Wang Y., Yuan F. (2018). The effects of public subsidies on emerging industry: An agent-based model of the electric vehicle industry. Technol. Forecast. Soc. Chang..

[15] Kong D., Xia Q., Xue Y., Zhao X. (2020). Effects of multi policies on electric vehicle diffusion under subsidy policy abolishment in China: A multi-actor perspective. Appl. Energy.

[16] Yang Y., Yang W., Chen H., Li Y. (2020). China’s energy whistleblowing and energy supervision policy: An evolutionary game perspective. Energy.

[17] Liu C., Huang W., Yang C. (2017). The evolutionary dynamics of China’s electric vehicle industry–Taxes vs. subsidies. Comput. Ind. Eng..

[18] Shin K., Yeo Y., Lee J.-D. (2020). Revitalizing the Concept of Public Procurement for Innovation (PPI) from a Systemic Perspective: Objectives, Policy Types, and Impact Mechanisms. Syst. Pract. Action Res..

[19] Li J., Jiao J., Tang Y. (2020). Analysis of the impact of policies intervention on electric vehicles adoption considering information transmission—based on consumer network model. Energy Policy.

[20] Oliveira G.D., Roth R., Dias L.C. (2019). Diffusion of alternative fuel vehicles considering dynamic preferences. Technol. Forecast. Soc. Chang..

[21] Majerova J. (2022). Cognitive Rationality and Sustainable Decision Based on Maslow’s Theorem: A Case Study in Slovakia. Cogn. Sustain..

[22] Kang Y., Chen J., Wu D. (2020). Research on Pricing and Service Level Strategies of Dual Channel Reverse Supply Chain Considering Consumer Preference in Multi-Regional Situations. Int. J. Environ. Res. Public Health.

[23] Janssen M.A., Jager W. (2001). Fashions, Habits and Changing Preferences: Simulation of Psychological Factors Affecting Market Dynamics. J. Econ. Psychol..

[24] Lachaab M., Ansari A., Jedidi K., Trabelsi A. (2006). Modeling preference evolution in discrete choice models: A Bayesian state-space approach. Quant. Mark. Econ..

[25] Nikas A., Lieu J., Sorman A., Gambhir A., Turhan E., Baptista B.V., Doukas H. (2020). The desirability of transitions in demand: Incorporating behavioural and societal transformations into energy modelling. Energy Res. Soc. Sci..

[26] Xiong H., Payne D., Kinsella S. (2016). Peer effects in the diffusion of innovations: Theory and simulation. J. Behav. Exp. Econ..

[27] Li D., Du J., Sun M., Han D. (2020). How conformity psychology and benefits affect individuals’ green behaviours from the perspective of a complex network. J. Clean. Prod..

[28] Mi L., Zhu H., Yang J., Gan X., Xu T., Qiao L., Liu Q. (2019). A new perspective to promote low-carbon consumption: The influence of reference groups. Ecol. Econ..

[29] Oryani B., Koo Y., Shafiee A., Rezania S., Jung J., Choi H., Khan M.K. (2022). Heterogeneous preferences for EVs: Evidence from Iran. Renew. Energy.

[30] Chakraborty D., Bunch D.S., Brownstone D., Xu B., Tal G. (2022). Plug-in electric vehicle diffusion in California: Role of exposure to new technology at home and work. Transp. Res. Part A Policy Pract..

[31] Lee J.H., Hardman S.J., Tal G. (2019). Who is buying electric vehicles in California? Characterising early adopter heterogeneity and forecasting market diffusion. Energy Res. Soc. Sci..

[32] Moon H., Park S.Y., Woo J. (2021). Staying on convention or leapfrogging to eco-innovation? Identifying early adopters of hydrogen-powered vehicles. Technol. Forecast. Soc. Chang..

[33] Fan R., Wang Y., Lin J. (2021). Study on Multi-Agent Evolutionary Game of Emergency Management of Public Health Emergencies Based on Dynamic Rewards and Punishments. Int. J. Environ. Res. Public Health.

[34] Luo M., Fan R., Zhang Y., Zhu C. (2020). Environmental Governance Cooperative Behavior among Enterprises with Reputation Effect Based on Complex Networks Evolutionary Game Model. Int. J. Environ. Res. Public Health.

[35] Shi Y., Han B., Zeng Y. (2020). Simulating policy interventions in the interfirm diffusion of low-carbon technologies: An agent-based evolutionary game model. J. Clean. Prod..

[36] Li F., Cao X., Ou R. (2021). A network-based evolutionary analysis of the diffusion of cleaner energy substitution in enterprises: The roles of PEST factors. Energy Policy.

[37] Xu J., Zhai J., Li F., Lv X. (2020). Research on Diffusion Mechanism of Green Innovation of Cloud Manufacturing Enterprises Based on BA Scale-Free Agglomeration Network Game. IEEE Access.

[38] Shi Y., Wei Z., Shahbaz M., Zeng Y. (2021). Exploring the dynamics of low-carbon technology diffusion among enterprises: An evolutionary game model on a two-level heterogeneous social network. Energy Econ..

[39] Cheng X., Long R., Chen H., Yang J. (2019). Does social interaction have an impact on residents’ sustainable lifestyle decisions? A multi-agent stimulation based on regret and game theory. Appl. Energy.

[40] Zino L., Ye M., Cao M. (2020). A two-layer model for coevolving opinion dynamics and collective decision-making in complex social systems. Chaos Interdiscip. J. Nonlinear Sci..

[41] Ye M., Zino L., Rizzo A., Cao M. (2021). Game-theoretic modeling of collective decision making during epidemics. Phys. Rev. E.

[42] Zeng Y., Dong P., Shi Y., Wang L., Li Y. (2020). Analyzing the co-evolution of green technology diffusion and consumers’ pro-environmental attitudes: An agent-based model. J. Clean. Prod..

[43] Fan R., Dong L. (2018). The dynamic analysis and simulation of government subsidy strategies in low-carbon diffusion considering the behavior of heterogeneous agents. Energy Policy.

[44] Wu B., Liu P., Xu X. (2017). An Evolutionary Analysis of Low-Carbon Strategies Based on the Government enterprise Game in the Complex Network Context. J. Clean. Prod..

[45] Lin J., Fan R., Tan X., Zhu K. (2021). Dynamic decision and coordination in a low-carbon supply chain considering the retailer's social preference. Socio-Econ. Plan. Sci..

[46] Safarzyńska K., Bergh J.C.V.D. (2017). Integrated crisis-energy policy: Macro-evolutionary modelling of technology, finance and energy interactions. Technol. Forecast. Soc. Chang..

[47] Huang X., Lin Y., Zhou F., Lim M.K., Chen S. (2021). Agent-based modelling for market acceptance of electric vehicles: Evidence from China. Sustain. Prod. Consum..

[48] Moore F.C., Lacasse K., Mach K.J., Shin Y.A., Gross L.J., Beckage B. (2022). Determinants of emissions pathways in the coupled climate—Social system. Nature.

[49] Adnan N., Nordin S.M., Rahman I., Amini M.H. (2017). A market modeling review study on predicting Malaysian consumer behavior towards widespread adoption of PHEV/EV. Environ. Sci. Pollut. Res..

